# Zerumbone mediates apoptosis and induces secretion of proinflammatory cytokines in breast carcinoma cell culture 

**DOI:** 10.22038/IJBMS.2021.58573.13012

**Published:** 2021-11

**Authors:** Muttiah Barathan, Kumutha Malar Vellasamy, Zaridatul Aini Ibrahim, Vanitha Mariappan, See Mee Hoong, Jamuna Vadivelu

**Affiliations:** 1 Department of Medical Microbiology, Faculty of Medicine, University of Malaya, Lembah Pantai, Kuala Lumpur 50603, Malaysia; 2 Department of Pharmacology, Faculty of Medicine, University of Malaya, Lembah Pantai, Kuala Lumpur 50603, Malaysia; 3 Center of Toxicology and Health Risk Studies (CORE), Faculty of Health Sciences, National University of Malaysia, Jalan Raja Muda Aziz, Kuala Lumpur 50300, Malaysia; 4 Department of Surgery, Faculty of Medicine, University of Malaya, Lembah Pantai, Kuala Lumpur 50603, Malaysia

**Keywords:** Apoptosis, Breast, Cytokine, Natural, Zerumbone

## Abstract

**Objective(s):**

To investigate the potential anti-breast cancer activity of zerumbone in regulating apoptotic mediators and cytokines in comparison with paclitaxel (positive control).

**Materials and Methods:**

In this study, assays such as viability, apoptosis, reactive oxygen species, cell cycle, DNA fragmentation, and cytokines were carried out on MCF-7 cells after treatment with zerumbone and paclitaxel.

**Results:**

The results showed that zerumbone demonstrated a higher (18-fold) IC_50 _value (126.7 µg/ml) than paclitaxel (7.29 µg/ml) in order to suppress proliferation and induce cell death of MCF-7. The cell cycle arrest at the G0/G1 phase and excessive intracellular ROS production during the *in vitro* zerumbone treatment indicated occurrence of apoptotic cell death although nuclear DNA fragmentation was not observed. The flow cytometer analysis of treated cells revealed secretion of proinflammatory cytokines suggesting the potential immunomodulatory activity of zerumbone.

**Conclusion:**

Although, zerumbone exhibited a higher IC_50_ value compared with paclitaxel yet its anticancer activity against MCF-7 cells is still parallel to paclitaxel hence zerumbone has the potential to be an antineoplastic agent in the treatment of breast cancer especially the luminal type A.

## Introduction

Breast cancer has been reported to be the most commonly found cancer of women worldwide (1). In 2020, more than 650,000 women expired from this cancer and that number is expected to rise (2). Breast cancer starts in the cells of the lobules (milk glands) or in the ducts around the lobules in which they can spread or metastasize to the other parts of the body. This usually occurs in the advanced stages of cancer, making it difficult to treat (3). Early detection and aggressive combination therapies that include surgery, chemotherapy, targeted biological agents, and/or radiation therapy are the current clinical practices for breast cancer treatment (4). Unfortunately, these therapies are accompanied by severe side effects and are not always effective as the cancer cells may acquire drug-resistant phenotype as well as recurrence of the tumor (5). One of the treatment approaches by oncologists to overcome these problems is by administrating an increased dosage of chemotherapeutic agents to cancer patients to attain effective anti-tumor response (6). However, this leads to several other adverse effects including worsened drug toxicity, impairment of natural immune system as well as increased exposure to bacterial infection. Therefore, there is a crucial need for innovations in cancer treatment to address the serious adverse effects and failure of chemotherapy (7).

Over 15 years, researchers have exploited various natural products derived from microbial, plant, and animal species to treat many types of infectious and non-infectious human diseases (8). Taxol and doxorubicin, both derived from plants, have demonstrated promising results in the treatment of cancer. Taxol from the bark of *Taxus brevifolia* (paclitaxel) presents one of the best chemotherapy drugs for various cancers such as ovarian, breast, melanoma, and other solid tumors (9). It is an anti-microtubule agent that inhibits cancer cell growth and proliferation (10). Meanwhile, doxorubicin an anthracycline antibiotic is most extensively used in treatment of brain, breast, and cervix cancers and Hodgkin’s lymphoma (11). This drug causes DNA damage to the proliferating cancer cells leading to cell growth inhibition (12). Several natural products can regulate cell death and secretion of proinflammatory mediators including cytokines in cancer cells. For instance, curcumin and japonicone A can inhibit inflammation and growth of cancer cells (13). Others have also reported that plant-derived anticancer drugs such as thymoquinone, etoposide, and irinotecan can induce cell death and apoptosis in cancer cells via various mechanisms including DNA cleavage mediated by topoisomerase I or II inhibition (14), cellular metabolism enzyme inhibition (15), mitochondrial permeabilization (16), autophagy inhibition (17) and tumor-induced angiogenesis inhibition (18).

**Figure 1:**
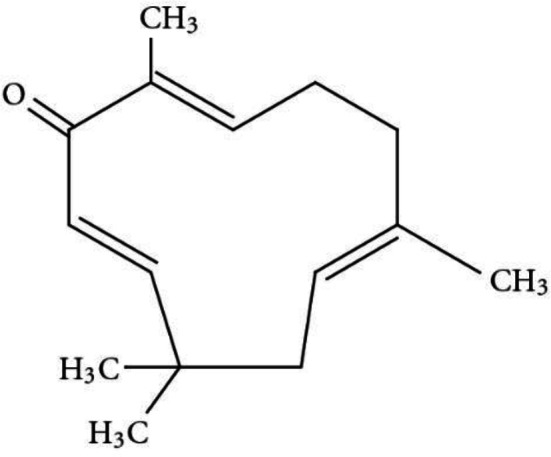
Structure of zerumbone. The figure was derived from Eid *et al*., 2019 (19)

Zerumbone (Figure 1), a compound derived from the wild shampoo ginger of the subtropical Zingiberaceae family, is traditionally used for pain relief. Lately, zerumbone has been proven for its anti-oxidant, and pro- as well anti-inflammatory properties (19). Additionally, recent publications demonstrated the anti-proliferative properties of zerumbone by inducing dose-dependent cell death and cell cycle arrest in many cancer cell types such as colon, breast, cervical, and liver (20), although its exact mechanism of action has not been entirely reported in comparison with clinically used drugs. Therefore, we studied the potential anticancer activity of zerumbone against luminal type A breast cancer cell line, MCF-7 cells, in comparison with paclitaxel, a clinically used antineoplastic drug to treat all types of breast cancers. Furthermore, we elucidated the possible mechanisms by investigating the apoptotic mediators and cytokines involved during *in vitro* anticancer treatment. 

## Materials and Methods

***Chemicals and reagents***

Zerumbone, paclitaxel, and other chemical reagents such as dimethyl sulfoxide (DMSO), MTT powder, agarose, and DNA ladder were obtained from Sigma-Aldrich, USA. 

***Normal and cancer cell lines***

MCF-7 cell line and non-tumorigenic epithelial cell line (MCF10A) were acquired from the American Type Culture Collection (Rockville, MD, USA). Dulbecco’s Modified Eagle Medium (DMEM) /F12 Ham’s medium supplemented with 10% of fetal bovine serum (FBS) (Sigma), 10 ng/ml of epidermal growth factor (Sigma), 10 μg/ml of insulin (Sigma), 0.5 mg/ml of hydrocortisone (Sigma), 100 ng/ml of cholera toxin (Sigma), 100 units/ml of penicillin (Sigma), and 100 μg/ml of streptomycin (Sigma) were used to grow MCF10A in a 37 °C humidified incubator with 5% CO2. Similarly, MCF-7 cells were propagated in an incubator in a flask containing high glucose DMEM medium supplemented with 10% fetal bovine serum, 2 mM L-glutamine, 100 units/ML of penicillin, and 100 μg/ml of streptomycin.

***Viability assay***

Viability of MCF-7 after zerumbone treatment and the half-maximal inhibitory concentration (IC_50_) of zerumbone were confirmed using a 3-(4,5-dimethylthiazol-2-yl)-2,5-diphenyltetrazolium bromide (MTT) assay. In brief, MCF-7 cells were grown (5 X 10^4^ cells/ml) in a 96-well plate for 24 hr and the medium was replaced followed by zerumbone treatment using various concentrations such as 200, 175, 150, 125, 100, 75, 50, and 25 μg/ml for 6, 12, 24, 48, and 72 hr, respectively. The results were obtained using a microplate reader at a wavelength of 570 nm, and the IC_50_ value of zerumbone was determined using the Graph pad software. The non-treated cells were incorporated as a negative control meanwhile paclitaxel was a positive control in these experiments from the range of 1 to 8 μg/ml. 

***Apoptosis assay***

Since the IC_50_ value of zerumbone and paclitaxel on MCF-7 cells was determined, next the apoptotic effect of that concentration of these natural products was investigated through a Tali® cytometer. Apoptosis Kit included Annexin V Alexa Fluor® 488 and propidium iodide (ThermoFisher, Waltham, MA, USA). Similar to viability assay, the MCF-7 cells were cultivated in 6-well plates followed by zerumbone and paclitaxel treatment for 48 hr. The cells were subjected to apoptosis assay and examined using a Tali® Image-Based Cytometer (ThermoFisher). Data analysis was carried out using FCS Express Research Edition software (version 4.03; De Novo Software, USA). The final output of these assays was projected in the percentage of each population: live, dead, and apoptotic cells in that particular cell culture after treatment with natural products. The visual result was also obtained whereby apoptotic cells were stained as green due by Annexin V Alexa Fluor® 488, dead cells were stained with red by PI, and green by Annexin V Alexa Fluor® 488, and live cells remained unstained.

***Cell cycle assay***

The cell cycle distributions of MCf-7 cells after zerumbone and paclitaxel treatment were investigated with the help of a BD BrdU FITC kit and a BD FACSCanto II flow cytometer (BD Biosciences, USA). The treated MCF-7 cells with zerumbone and paclitaxel were stained with 10 μM bromodeoxyuridine (BrdU) for 30 min and 20 μl anti-BrdU-FITC for 20 min as well as with 2.5 μl 7-amino-actinomycin D (7-AAD) for 15 min after the 48 hr treatment. The data were acquired using a flow cytometer and analyzed using the FlowJo v7.6.5 64-bit software (Treestar, Ashland, USA). 

***Reactive oxygen species (ROS) assay***

To further confirm the anticancer activity of zerumbone, the release of cellular ROS was investigated using a DCFDA / H2DCFDA cellular ROS assay kit (Mitoscience/Abcam, Cambridge, UK) in MCF-7 cell culture after zerumbone treatment for 48 hr. The cellular ROS was detected by fluorescence spectroscopy, using a Varioskan Flash microplate reader (Thermo-Scientific, USA) at 495 nm (excitation) and 529 nm (emission). 

***DNA fragmentation assay***

DNA fragmentation assay was investigated on the DNA of MCF-7 after zerumbone treatment to check whether zerumbone could induce nuclear damage in MCF-7 cells. The DNA was extracted using a commercial QIAamp DNA mini kit (Qiagen, Valencia, CA, USA). The extracted DNA of treated MCF-7 and non-treated MCF-7 were electrophoresed on 1.4% (w/v) agarose gel for 1 hr and imaged using a Gel Doc XR gel documentation system (Bio-Rad, UK). 

***Th1/Th2/Th17 cytokines assay***

To check whether zerumbone could induce the release of proinflammatory cytokines and cause inflammation related to cell death, a commercially available BD Cytometric Bead Array (CBA) Human Th1/Th2/Th17 Cytokine kit (BD Biosciences, San Jose, CA, USA) was used to measure the secretion levels of various cytokines IL-2, IFN-γ, TNF-α, IL-6, IL-10, IL-4, and IL-17A with help of FACSCanto II (BD Biosciences) and analyzed using the FCAP array software (BD Biosciences). 

***Statistical analysis***

Each experiment was repeated at least thrice and analyzed in mean ± standard deviation (SD) format. The statistical comparisons between each group were performed using a two-tailed unpaired Student’s t-test and a one-way or two-way analysis of variance with Bonferroni’s *post hoc* test (more than 2 groups). Differences were considered significant at *P<0.05, **P<0.01, and ***P<0.001.

## Results

***Cytotoxicity level of zerumbone***

**Figure 2:**
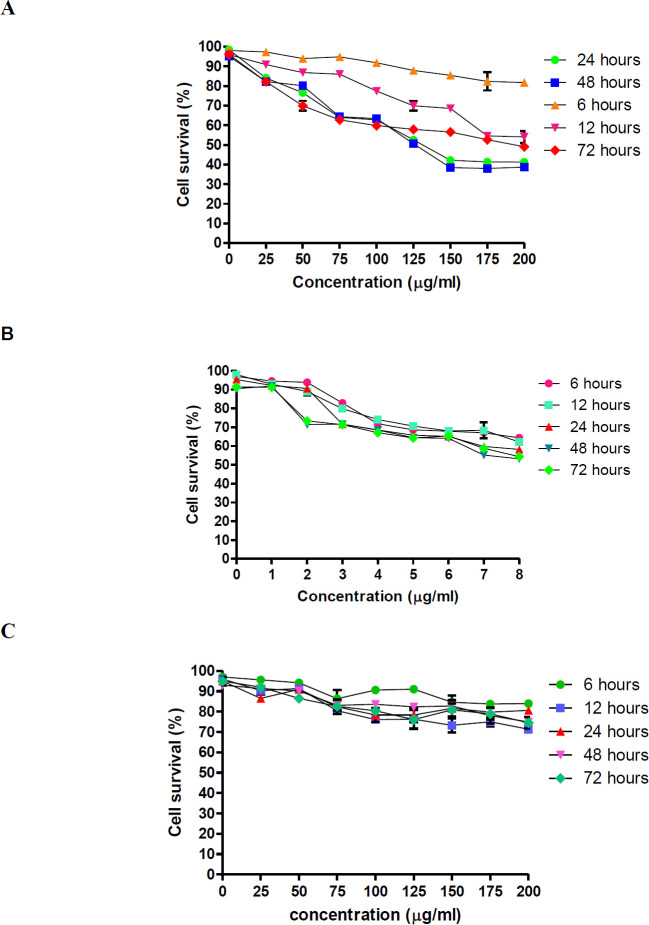
(A) Dose-dependent survival of MCF-7 cells treated with zerumbone as determined by MTT assay. A higher reduction of MCF-7 cell growth was found at a higher concentration of zerumbone. (B) Dose-dependent survival of MCF-7 cells treated with paclitaxel as determined by MTT assay. Different treatment periods exhibit differential sensitivities to paclitaxel treatment *in vitro*. Dose-response curves were obtained using a logistic nonlinear regression analysis model. (C) Dose-dependent survival of MCF-10A cells against zerumbone as determined by MTT assay. Data are presented as mean ± standard deviation (SD) from duplicate samples of at least three independent experiments

Cytotoxicity of zerumbone and paclitaxel against MCF-7 cells was tested using various concentrations ranging from 25 to 200 µg/ml for zerumbone and 1 to 8 µg/ml for paclitaxel at different time points: 6, 12, 24, 48, and 72 hr. The cytotoxicity of zerumbone and paclitaxel on MCF-7 was measured by the number of viable cells containing NAD(P)H-dependent oxidoreductase enzymes in the cell culture (21) and it was compared with untreated cells. Based on this analysis, both drugs demonstrated a dose-dependent induction of cell death at different time points. As the concentration of these compounds increased, a significantly sharp reduction of MCF-7 cell survival was observed (*P*<0.01). MCF-7 cells’ viability decreased significantly with increasing zerumbone and paclitaxel concentrations (Figures 2A&B). Nearly 20% of cells were found inhibited at the lowest concentration of zerumbone (25 µg/ml), and as the concentration of zerumbone increased, a steady decrease in the number of MCF-7 cells that survived was observed, nearly 50% of cells were inhibited at the highest concentration of zerumbone (200 µg/ml). A similar trend was found at 48 hr of treatment point. Besides, it was found that treating MCF-7 cells with zerumbone for 48 hr exhibited a lower IC_50_ value (126.7 µg/ml) compared with 24 hr (127 µg/ml). Meanwhile, treating the MCF-7 cells with zerumbone for 6, 12, and 72 hr needed much higher IC_50_ than 24 and 48 hr. However, due to lower IC50 of zerumbone upon MCF-7 cells at the treatment period of 48 hr compared with 24 hr, 48 hr was selected for future experiments. Meanwhile, paclitaxel inhibited MCF-7 cells by 50% with 7.29 µg/ml and 7.43µg/ml in 24 and 48 hr, respectively. On the other hand, treating cells at 72 hr exhibited slightly higher IC_50_ (8.01 µg/ml). Approximately 48% and 10% of MCF-7 cells were found to inhibit at the highest (8 µg/ml) and lowest concentrations of paclitaxel (1 µg/ml) after 24 hr of treatment. A similar trend was found at 48 and 72 hr of treatment point. However, treatment points of 6 and 12 hr were found to exhibit higher IC50 compared with others. For paclitaxel, a treatment period of 24 hr was selected due to the lower IC_50_ needed to inhibit the growth of MCF-7 cells. Moreover, it was found that a much lower IC_50_ of paclitaxel was needed to suppress the viability of MCF-7 at 48 hr. In addition, the IC_50_ of zerumbone did not have a cytotoxic effect on MCF10A, normal breast epithelial (Figure 2C), thus maintaining its highly selective properties towards tumor cells (22).

***Zerumbone induces apoptosis in MCF-7 cells***

**Figure 3:**
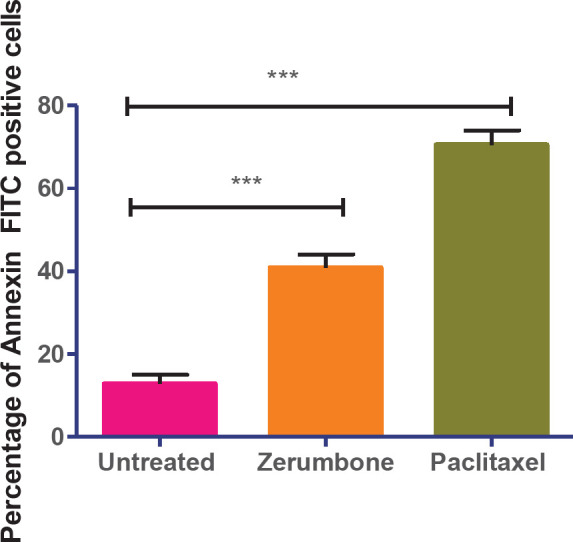
Zerumbone induces apoptosis in MCF-7 cells after 48 hr. The data represent the means ± standard deviations (SDs) of 3 independent tests. Statistical analysis is defined as significant if *P<0.05, **P<0.01, and ***P<0.001

Since there were no zerumbone and paclitaxel effects on MCF10A, additional experiments were conducted only on the MCF-7 cells. The fluorometric results demonstrated that zerumbone (126.7 µg/ml, IC_50_) induced 37% of apoptotic cells after 48 hr compared with untreated cells (16% of apoptotic cells) (*P*<0.001), indicating the occurrence of *in vitro* cell death in MCF-7 cells. Meanwhile, MCF-7 cells had a significant 2-fold (75% of apoptotic cells) increase in the induction of apoptosis during the treatment of paclitaxel (7.43 µg/ml, IC_50_) (*P*<0.001) as compared with zerumbone (Figure 3).

***Zerumbone induces G0/G1 cell cycle arrest***

**Figure 4:**
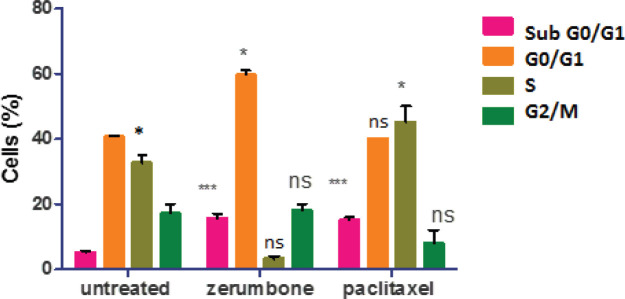
The quantitative analysis indicated zerumbone arrested cell at the G0/G1 and paclitaxel arrested at the S phases. The data represent the mean ± SD of 3 independent tests. Statistical analysis is defined as significant if *P<0.05, **P<0.01, and ***P<0.001

In the analysis, the untreated MCF-7 cells were found to actively proliferate whereby 43% of cells were found to augment in the G0/G1 phase, followed by 36% at S, and 16% at G2/M phases. However, following treatment with zerumbone (126.7 µg/ml, IC_50_), the cells were observed to enter cell cycle arrest and formation of apoptotic cells. It was found that a large proportion of zerumbone treated MCF-7 cells arrested at the G0/G1 phase (62%), hence the decrease in the cell population at the G2/M (16%) and S (5%) phases were seen. The occurrence of apoptosis in MCF-7 cells after zerumbone treatment was further confirmed by aggregation of MCF-7 cells (17% of the total cell population underwent apoptosis) in the sub-G0/G1 phase compared with 5% of apoptotic cells in untreated MCF-7. In contrast, paclitaxel (7.43 µg/ml, IC_50_) induced approximately 23% of total cells found at the G0/G1 phase, and approximately 45% of cells were observed to enter the cell cycle arrest at S phase as well as only 8% of cells at G2/M phase. The flow cytometric analysis also demonstrated significant augmentation of apoptosis of 17% of the cells following treatment with paclitaxel (7.43 µg/ml, IC_50_). These findings demonstrate the ability of zerumbone to induce cell cycle arrest at the G/G1 phase and prevent cell proliferation in MCF-7 cell culture (Figure 4).

***Release of cellular reactive oxygen species (ROS) from zerumbone treated cells***

**Figure 5:**
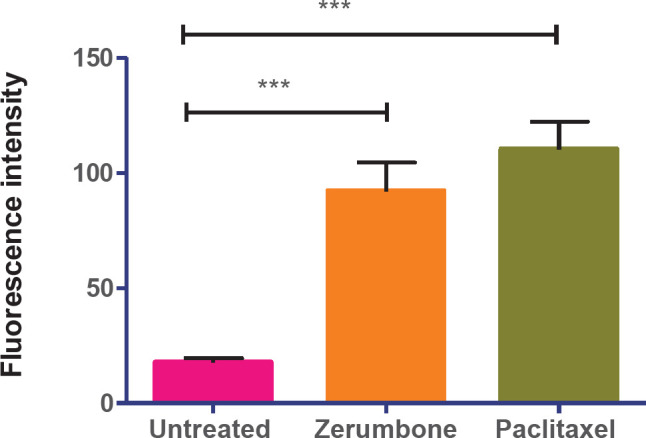
Reactive oxygen species (ROS) generation in treated MCF-7 cells. Fluorescence intensity after the treatment of IC_50_ of zerumbone and paclitaxel. The graph shows that zerumbone induces cellular ROS almost similar to paclitaxel. The data represent the mean ± standard deviation (SD) of 3 independent experiments. Statistical analysis is defined as significant if *P<0.05, **P<0.01, and ***P<0.001

The fluorometric analysis displayed that zerumbone (42 counts per second (cps) and paclitaxel (65 cps) induced ROS production in MCF-7 cells culture meanwhile untreated cells exhibited a significantly low production of ROS (15 cps) suggesting that these plant natural products could induce oxidative stress and damage the cancer cells (Figure 5). However, there was no difference in the ROS levels between the two compounds when further statistical comparisons were made between them.

***Zerumbone induces DNA fragmentation in treated MCF-7 cells***

**Figure 6:**
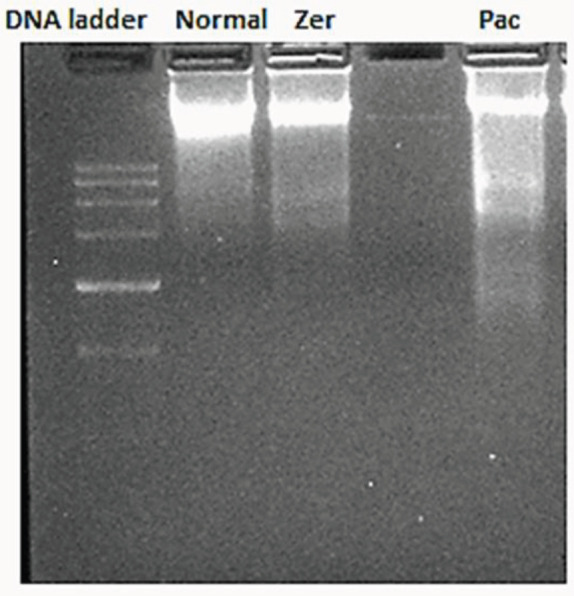
A clear DNA fragmentation in MCF-7 treated with paclitaxel. zerumbone did not induce a significant pattern of DNA fragmentation compared with paclitaxel. Faint DNA fragments were visible in the lane of zerumbone treated MCF-7 cells. Lane 1: 1000 bp DNA ladder, Lane 2: DNA from untreated MCF-7 cells, Lane 3: DNA from IC50 of zerumbone treated MCF-7 cells, Lane 4: DNA from IC_50_ of paclitaxel treated MCF-7 cells

Zerumbone was seen to suppress the proliferation of MCF-7 at the G0/G1 phase and induce apoptosis in MCF-7 at 48 hr; however, a faint DNA laddering was observed in cells treated with zerumbone, compared with paclitaxel, clear and defined DNA laddering was observed (Figure 6).

***Differential of Th1/Th2/Th17 cytokine release in the MCF-7 cells after zerumbone treatment***

**Figure 7:**
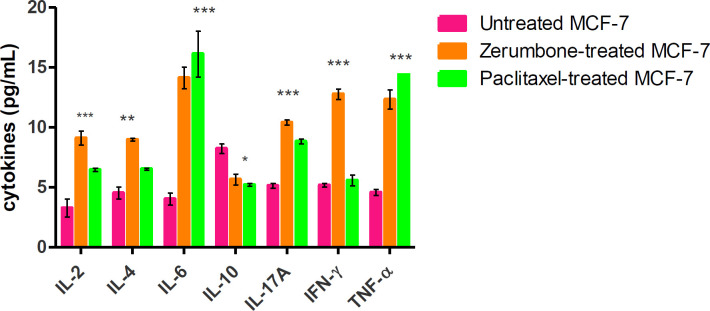
Zerumbone and paclitaxel treatment triggers secretion of proinflammatory cytokines in MCF-7 cells. A significant increase in secretion of IL-6, TNF- α, and IFN-γ upon treatment of naturally occurring phytochemicals upon MCF-7 cells. The data represent the mean ± standard deviation (SDs) of 3 independent experiments. Statistical analysis is defined as significant if *P<0.05, **P<0.01, and ***P<0.001, ns: non-significant

Flow cytometric analysis demonstrated a mixed expression pattern of pro-inflammatory cytokines (IL-6, IL-2, TNF-α, IFN-γ, and IL-17A) and anti-inflammatory cytokines (IL-10 and IL-4). A low level of IL-2 (3.5 pg/ml), IL-6 (4 pg/ml), TNF-α (4.2 pg/ml), IFN-γ (4.3 pg/ml), and IL-17A (5.7 pg/ml) were found to be secreted in the supernatant of untreated MCF-7 cells (Figure 7). Also, it was found that IL-10 (7.9 pg/ml) was highly secreted followed by and IL-4 (4.8 pg/ml)) in the untreated MCF-7 culture. Following treatment of the MCF-7 cells with zerumbone (126.7 µg/ml, IC_50_) led to up-regulation of cytokines such as IL-6 (14.4 pg/ml), IFN-γ (12.3 pg/ml), TNF-α levels (10 .7 pg/ml), IL17A (10 pg/ml) and IL-2 (8.5 pg/ml) were found significantly expressed as well (*P*<0.01). Besides, a significant decrease in IL-10 (5 pg/ml) was found in the culture supernatant of MCF-7 after zerumbone treatment yet IL-4 was found to be up-regulated (IL-4 (8.5 pg/ml) compared with non-treated MCF-7 (*P*<0.00)). In addition, MCF-7 cells treated with paclitaxel (7.43 µg/ml, IC_50_) demonstrated no significant difference in the trend of expression of the pro- and anti-inflammatory cytokines as compared with zermbone. Values found were TNF-α (15.5 pg/ml), IL-6 (15 pg/ml) (*P*<0.001), IL-2 (9.4 pg/ml), IL-17A (8.3 pg/ml), and IFN-γ (5.6 pg/ml) (*P*<0.01). Expression of IL-10 (4.8 pg/ml) was reduced, similar to the trend seen using zerumbone. However, there was a significant increase in the expression of IL-4 (9.4 pg/ml) (*P*<0.05). 

## Discussion

Zerumbone has been shown to induce anti-inflammatory properties in microglial cells (a type of macrophage cell) by inhibiting the mitogen-activated protein kinase (MAPK) pathway, proposing the role of zerumbone as a neuroprotector, besides zerumbone is also reported to have anticancer, pro-inflammatory, and chemoprevention against various cancers (19). Interestingly, zerumbone shows no cytotoxic effect towards normal breast cancer cells suggesting that zerumbone is safe to be used in treatment of breast cancer since zerumbone specifically kills breast cancer cells. Hence, zerumbone is investigated for its potential as an antineoplastic drug against paclitaxel for the treatment of breast cancer. A recent report has described findings that demonstrated a similar pattern of MCF-7 cell killing by paclitaxel (23), which was used as a positive control in a comparison study of the ionic liquid extract of Graviola fruit (IL-GFE) (24). Although, the concentration of zerumbone needed to inhibit the growth of MCF-7 was higher compared with paclitaxel, that concentration did not have any cell-killing effects on normal breast cells (MCF10A), which indicates the potential role of zerumbone as an antineoplastic agent. The data presented in this study suggest high selective action of zerumbone against cancer cells. We also investigated the anticancer properties of zerumbone in inducing apoptosis, ROS, and DNA fragmentation as well as cytokine production in the MCF-7 cell line.

In this study, the occurrence of apoptosis in zerumbone and paclitaxel treated MCF-7 cells was evaluated based on the loss of membrane asymmetry which directly correlates with the appearance of phosphatidylserine (PS) on the surface of apoptotic cells which has a strong affinity towards Annexin V (25). It was found that 75% of MCF-7 cells treated with paclitaxel underwent significant apoptosis as compared with only 37% of cells treated with zerumbone. This is demonstrated that perhaps paclitaxel played a role in inducing the early stage of apoptosis on MCF-7 cells whereas zerumbone induced the mid-stage of apoptosis as an alert of the cell cycle of MCF-7 post-treatment was observed (26). Occurrence of apoptosis may lead to activation of complex signaling cascades such as loss of membrane asymmetry, cleavage of anti-apoptotic Bcl-2 family proteins, caspase activation, mitochondrial transmembrane potential, cytochrome C release, alert in cell cycle, nuclear fragmentation, and apoptotic body formation (27). Although events of apoptosis were found in untreated MCF-7 cells, this may have probably been due to the normal development and aging of cells (28). 

In the cell cycle assay using FACS, apoptotic cells were evident in the sub G0/G1 peak (apoptotic cells) using both IC_50_ of zerumbone and paclitaxel (29). In addition, after 48 hr of treatment, the cell cycle arrest (antitumor) effect of zerumbone on MCF-7 occurred at the G0/G1 phase whereas paclitaxel was arrested at the S phase. In summary, paclitaxel was found to induce cell arrest at the S phase indicating a reduction in the synthesis or replication of DNA leading to damage of intracellular DNA in the cancer cells resulting in cell death (30). On the other hand, zerumbone may inhibit cancer cells in the development of essential organelles and proteins thus halting the progression of tumorigenesis and apoptosis (31, 32). Therefore, given these findings, further studies should be conducted to further evaluate zerumbone’s potential as a new candidate for anticancer therapy.

**Figure 8:**
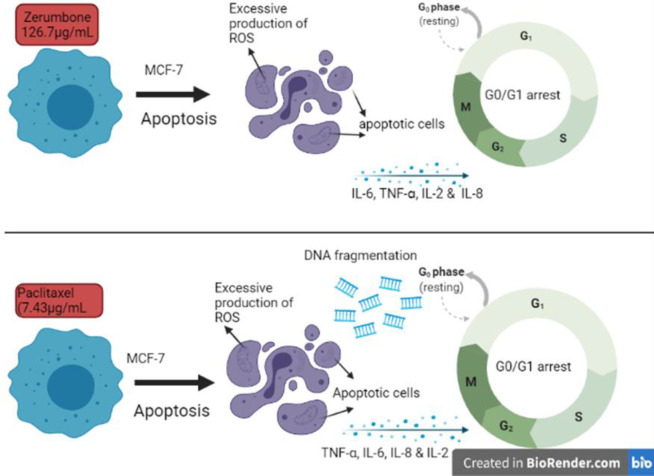
Potential anticancer mechanism behind zerumbone and paclitaxel treated MCF-7 cells such as formation of reactive oxygen species (ROS), apoptotic cells, and pro-inflammatory cytokines along with cell cycle arrest at G0/G1 phase

In the reactive oxygen species (ROS) assay using a fluorescence assay, higher levels of cellular ROS levels were released by the zerumbone treated cells similar to the paclitaxel treated MCF-7 culture. This excessive ROS production indicates that MCF-7 cells underwent oxidative stress-related cell death after 48 hr of treatment using zerumbone and paclitaxel (33). The oxidative stress-related cell death targets damage to cellular proteins, nucleic acids, and organelles specifically to mitochondrial-related cell death (34). Several studies have linked the secretion of ROS which induces inflammation to eventual activation of the secretion of cytokines responsible for inflammation such as IL-1 β, IL-6, and TNF-α through certain molecular pathways such as tumor necrosis factor receptor (TNFR) signaling and ROS-dependent cell death pathway. The present study exhibits that zerumbone has almost similar anticancer properties as the positive control, paclitaxel, whereby application of zerumbone on MCF-7 cell culture has induced cell apoptosis with help of mitochondrial ROS and pro-inflammatory cytokine-signaling pathway (Figure 8). It has been mentioned that antineoplastic drugs induced damage to the cancer cells, this makes the cancer cells release adenosine triphosphate (ATP) leading to the release of inflammatory cytokines and the formation of an inflammasome. Finally, this will lead to immunogenic cell death whereby the increased activity of cytotoxic T cells against tumor cells can be found during the treatment. Based on our analysis, zerumbone and paclitaxel have the potential to induce immunogenic cell death however further studies are needed to confirm this mechanism (35).

However, when we investigated whether zerumbone can also induce DNA fragmentation, zerumbone did not induce complete DNA fragmentation and DNA damage in MCF-7 cells after 48 hr of treatment unlike treatment with paclitaxel culture which showed clear DNA fragmentation following 24 hr of treatment. The occurrence of DNA fragmentation demonstrates the nuclear damage leading to late stage of apoptosis in MCF-7 cells (36). This is suggesting that paclitaxel may trigger cell death in breast carcinoma cells by inducing DNA damage, a late phase of apoptosis (37). It has been speculated that zerumbone induced slight nuclear fragmentation, it probably did not induce late-stage apoptosis at given IC_50_ and time point (38). However, a further experiment is needed to endorse this speculation.

 In the cytokine assay, release of different proinflammatory cytokines from the MCF-7 cells following treatment with zerumbone and paclitaxel was detected using FACS. Higher levels of TNF-ɑ than other proinflammatory cytokines were released from MCF-7 cells after zerumbone treatment. This suggests that TNF-ɑ may lead to the initiation of the inflammatory signaling pathway for apoptosis (39). Similarly, paclitaxel has also been shown to induce more pro-inflammatory cytokines as compared with anti-inflammatory cytokines. Cytokines such as IL-6, IL-8, and IL-2 were found to be up-regulated as well in culture supernatants of MCF-7 following treatment with zerumbone and paclitaxel, this will further contribute to promoting inflammation that will eventually activate the host immune response against tumor (40). On the other hand, zerumbone and paclitaxel treatment on MCF-7 cells led to a decrease of cytokines such as IL-10 and IL-17A in the culture supernatant compared with untreated MCF-7 cells, probably due to a higher level of secretion of proinflammatory cytokines that may suppress the expression of anti-inflammatory cytokines. A report has also shown that suppression of tumor cell killing T cells due to the release of IL-10 could lead to tumor growth (41, 42). In summary, zerumbone and paclitaxel treatment on MCF-7 cells induced up-regulation of proinflammatory cytokines and excessive inflammation, this could probably be due to the release of danger signals from zerumbone and paclitaxel treatment. This eventually activates an antitumor immune response against cancer cells (43, 44). Several other reports also have shown the ability of natural products such as curcumin (45) and doxorubicin (46) to induce immunological cell death in treated cancer cells. Hence, this suggests that zerumbone can be considered as well for further study on anticancer therapeutic potential using *in vivo* models.

## Conclusion

Taking into account the low toxicity and anti-proliferative efficiency of naturally occurring zerumbone, it might have a bright prospect for development similar to the commercially available anticancer drug, paclitaxel. In summary, our results support that zerumbone treatment induces* in vitro* cell death in breast carcinoma cells through up-regulation of ROS that could facilitate apoptosis through the mitochondria-mediated cell death, cell arrest at G0/G1 phase, and up-regulation of proinflammatory cytokines which may indicate activation of potential immunomodulatory activity. The previous data also suggested that zerumbone has a cytotoxic effect on MCF-7 cells, however, it did not explain molecular mechanisms in the regulation of cytokines. This set of data could further help us to develop zerumbone as a promising novel antineoplastic drug. 

## Funding Source

This piece of work was financed by Fundamental Research Grant Scheme (FRGS) (FP109-2019A), a research grant obtained from the Ministry of Education (MOE), Malaysia for a study entitled “anticancer activity of hyperforin against breast cancer cells: identification of cell death and autophagy pathways”. However, the funder has no role in this study. Muttiah BARATHAN was given financial support by the University of Malaya Student Financial Aid by the Institute of Research Management & Services (IPPP), University of Malaya, Malaysia.

## Authors’ Contributions

MB: Conceptualization, methodology, formal analysis, writing the original draft, and visualization. KMV: Conceptualization, methodology, writing, review, and editing. ZAI, AKZ: Writing, review, and editing. VM, SMH, JV: Conceptualization and methodology. 

## Financial Disclosure

The funder has agreed to disclose the findings. 

## Conflicts of Interest

There are no potential conflicts of interest reported by any of the authors.
